# Health-related quality of life and glycaemic control among adults with type 1 and type 2 diabetes – a nationwide cross-sectional study

**DOI:** 10.1186/s12955-019-1212-z

**Published:** 2019-08-14

**Authors:** Maria Svedbo Engström, Janeth Leksell, Unn-Britt Johansson, Sixten Borg, Bo Palaszewski, Stefan Franzén, Soffia Gudbjörnsdottir, Katarina Eeg-Olofsson

**Affiliations:** 1 Department of Molecular and Clinical Medicine, Institute of Medicine, University of Gothenburg, Sahlgrenska Academy, SE-405 30 Gothenburg, Sweden; 2 School of Education, Health and Social Studies, Dalarna University, SE-791 88 Falun, Sweden; 3 Department of Medical Sciences, Clinical Diabetology and Metabolism, Uppsala University, SE-751 85 Uppsala, Sweden; 4 Sophiahemmet University, SE-114 86 Stockholm, Sweden; 5 Department of Clinical Sciences and Education, Karolinska Institutet, Södersjukhuset, SE-118 83 Stockholm, Sweden; 6 Department of Clinical Sciences in Malmö, Health Economics Unit, Lund University, Medicon Village, SE-223 81 Lund, Sweden; 7 Department of Data Management and Analysis, Region Västra Götaland, Gothenburg, Sweden; 8 Register Center Västra Götaland, SE-413 45, Gothenburg, Sweden; 9 Sahlgrenska University Hospital, SE-413 45 Gothenburg, Sweden

**Keywords:** Diabetes mellitus, type 1, Diabetes mellitus, type 2, Health-related quality of life, SF-36, Cross-sectional study

## Abstract

**Background:**

Health-related quality of life and glycaemic control are some of the central outcomes in clinical diabetes care and research. The purpose of this study was to describe the health-related quality of life and assess its association with glycaemic control in adults with type 1 and type 2 diabetes in a nationwide setting.

**Methods:**

In this cross-sectional survey, people with type 1 (*n* = 2479) and type 2 diabetes (*n* = 2469) were selected at random without replacement from the Swedish National Diabetes Register. Eligibility criteria were being aged 18–80 years with at least one registered test of glycated haemoglobin (HbA_1c_) the last 12 months. The generic 36-item Short Form version 2 (SF-36v2) was answered by 1373 (55.4%) people with type 1 diabetes and 1353 (54.8%) with type 2 diabetes.

**Results:**

Correlation analyses showed weak correlations between scores on the SF-36v2 and glycaemic control for both diabetes types. After the participants were divided into three groups based on their levels of HbA_1c_, multivariate regression analyses adjusted for demographics, other risk factors and diabetes complications showed that among participants with type 1 diabetes, the high-risk group (≥70 mmol/mol/8.6%) had statistically significantly lower means in five out of eight domains of the SF-36v2 and the mental component summary measure, as compared with the well-controlled group (< 52 mmol/mol/6.9%). Among the participants with type 2 diabetes, the high-risk group had the lowest statistically significantly means in seven domains and both summary measures.

**Conclusions:**

Among people with type 1 and type 2 diabetes, adults with high-risk HbA_1c_ levels have lower levels of health-related quality of life in most but not all domains of the SF-36v2. This finding was not explained by demographics, other risk factors, or diabetes complications. The weak individual-level correlations between HRQOL scores and levels of glycaemic control argues for the need to not focus exclusively on either HbA_1c_ levels or HRQOL scores but rather on both because both are important parts of a complex, life-long, challenging condition.

**Electronic supplementary material:**

The online version of this article (10.1186/s12955-019-1212-z) contains supplementary material, which is available to authorized users.

## Introduction

Health-related quality of life (HRQOL) together with glycaemic control as measured by glycated haemoglobin (HbA_1c_) levels are some of the central outcomes in clinical diabetes care and research [1–8]. Through a suggested bidirectional relationship, diabetes and self-management demands might diminish HRQOL, possibly leading to worse glycaemic control and complications [3].

The medical outcomes study health survey short form (SF)-36 is a widely used generic HRQOL measurement tool recommended for comparisons and descriptions across subgroups with the same or different diseases. The RAND-36 has equivalent items but is scored differently [2, 4, 9, 10]. Cross-sectional associations between either the SF-36 or the RAND-36 and HbA_1c_ levels in adults have been addressed in a few studies of people with type 1 diabetes [11–13] and more commonly in studies of people with type 2 diabetes [14–20]. There are also studies of both groups combined [21, 22]. However, there have been inconsistent results. Previous studies have presented varying degrees of support both for [13, 14, 17, 19, 22] and against a relationship between HRQOL and glycaemic control [11–13, 15–18, 20, 21].

As a generic measure of HRQOL, the SF-36 has been criticized for not being specific enough for diabetes-related aspects [4, 9, 10, 23]. However, unlike diabetes-specific tools, a generic measure adds the potential to be able to relate the results to the overall population and to other health conditions. Among generic measures, the SF-36 is widely used in diabetes research and has been suggested as a reasonable choice in diabetes research as well as for application in diabetes care, with reference to the inclusion of a broad range of relevant aspects and supporting evidence for measurement quality [9, 10, 24, 25]. Furthermore, generic HRQOL in people with diabetes as measured by the SF-36 or the RAND-36 has been suggested to be a marker for mortality [26–28]. Despite the critique, it is suggested important to further research generic HRQOL in people with diabetes using the SF-36 [9].

Diabetes care continuously advances with new medical treatments, technical aids for insulin administration and the continuous monitoring of glucose levels, and skills facilitating self-management [1, 7, 29, 30]. Despite previous studies, there is a lack of updated data on HRQOL in people with diabetes. The aim of this study was thus to describe the HRQOL as measured by the SF-36 questionnaire and assess associations of that HRQOL and glycaemic control in adults with type 1 and type 2 diabetes in a nationwide setting with current diabetes care.

## Participants and methods

### Sample and data collection

In this cross-sectional postal survey, 2479 people with type 1 diabetes and 2469 with type 2 diabetes were selected at random without replacement from the Swedish National Diabetes Register (NDR), a nationwide quality register for diabetes care. In 2015, the NDR had above 95% coverage for people with type 1 diabetes and above 90% for people with type 2 diabetes. In total, there were about 40,000 people with type 1 diabetes and about 347,000 with type 2 diabetes registered in the NDR. Eligibility criteria were patients registered in the NDR during the period from September 30th 2014 to October 1st 2015, being alive, aged 18–80 years and having at least one HbA_1c_ level registered during the last 12 months. With these inclusion criteria there were 29,245 people with type 1 diabetes at hospital out-patient clinics and 208,852 people with type 2 diabetes at primary health care centres eligible for recruitment.

In October 2015, the SF-36v2 and a prepaid return envelope were sent by mail together with a newly developed diabetes-specific questionnaire. Data about the diabetes-specific questionnaire are reported elsewhere [31–33]. Non-responders received one reminder including the same material after 30 days. In total, 1373 (55.4%) people with type 1 diabetes and 1353 (54.8%) with type 2 diabetes answered the SF-36v2.

Clinical data (diabetes type defined by clinical diagnosis, diabetes duration, HbA_1c_ level, medical treatment, physical activity level, cardiovascular risk factors, and complications) and demographic data were obtained from the NDR. The clinical data collected from the NDR are registered in the NDR because of their important roles for high quality diabetes care.

### SF-36 version 2

SF-36 is a 36-item self-administered generic HRQOL questionnaire from the medical outcomes study. The SF-36 is internationally established with support for its validity and reliability [33, 34]. We used the SF-36 version 2 (SF-36v2) standard form in Swedish. An eight-domain profile is generated. The eight domains are physical functioning (PF); role-physical (RP), i.e., role limitations due to physical health problems; bodily pain (BP); general health (GH); vitality (VT); social functioning (SF); role-emotional (RE), i.e., role limitations due to mental health; and mental health (MH). The domains are scored from 0 to 100. The domains are aggregated in the Physical Component Summary (PCS) and the Mental Component Summary (MCS) measures, reported as norm-based *T*-scores. *T*-scores are standardized to the 2009 US general population with a mean of 50 and a standard deviation of 10. The average range for groups is a mean *T*-score between 47 and 53. Higher scores represent better HRQOL [33, 34].

### Statistical analysis

All analyses were performed separately for participants with type 1 and type 2 diabetes. Descriptive statistics are presented as the means and standard deviations for normally distributed continuous variables, the median and interquartile range for skewed distributions, or number and percentages for categorical variables. The descriptive statistics for each variable are based on non-missing observations. For variables given as percentage, the denominator is defined as all individuals with non-missing observations.

The SF-36v2 data were scored according to the manual using licensed software from QualityMetric Inc. To examine associations between SF-36v2 scores and HbA_1c_ levels, we first used Spearman’s rank correlation with HbA_1c_ as a continuous variable. For the group-level analysis, HbA_1c_ was considered a categorical variable and was divided into the following three clinically relevant groups with differing levels of glycaemic control and therefore differing levels of the risk of diabetes complications according to international and Swedish treatment guidelines: well-controlled (< 52 mmol/mol/6.9%), sub-optimal (52–69 mmol/mol/6.9–8.5%), and high-risk (≥70 mmol/mol/8.6%). Between the HbA_1c_ groups, the data balance and the deviation from the means in the clinical and demographic data were examined using the standardized mean difference.

Unadjusted and adjusted multivariate regression analyses were used to calculate the least square mean estimates and 95% confidence intervals for the SF-36v2 domains and summary measures in the three HbA_1c_ groups. The observations in each SF-36v2 domain and summary measure were modelled using a linear model with fixed effects for the HbA_1c_ group (exposure), age, sex, diabetes duration, body mass index (BMI), systolic blood pressure (SBP), LDL cholesterol level, micro and macro albuminuria, estimated glomerular filtration rate (eGFR), retinopathy, smoking status, physical activity level, receipt of antihypertensive and lipid lowering treatments, previous coronary heart disease (CHD) and previous stroke. The results are presented as least square mean estimates with 95% confidence intervals. The amount of missing data was 0% for demographics, 7.2% for clinical data (range 0–36.5%), and 1.7% for SF-36 domains (range 0–3.3% for individual dimensions). Missing data were imputed ten times using multiple chained equations. The analyses were performed separately for each imputed data set, and the results were subsequently combined using Rubin’s rules.

A significance level of 5% was used throughout; no allowance was made for multiplicity of statistical tests.

SAS 9.4 and R 3.4.4 were used for the clinical and demographic descriptive statistics and the correlation and regression analyses.

### Ethical considerations

The study conforms to the Declaration of Helsinki and was approved by the Regional Ethical Review Board in Gothenburg, Sweden (No. 029–15, T600–15). The letter to the participants informed them about the study’s purpose, the voluntary nature of their participation, the confidentiality measures and methods of handling of their personal data, the NDR, contact details, and the right to end participation. Participants gave their informed consent.

## Results

The clinical and demographic characteristics of the responders separated by diabetes type and HbA_1c_ level are presented in Table 1. Among those with type 1 diabetes, 50.3% of the responders were men, the average age was 48.6 years, the average diabetes duration was 24.7 years, and the average HbA_1c_ level was 62 mmol/mol (7.8%). Among those with type 2 diabetes, the corresponding numbers were 60.8% men, average age of 66.6 years, average duration of 9.4 years, and average HbA_1c_ level of 53 mmol/mol (7.0%) (Table 1). The crude means and standard deviations for the SF-36v2 domains for participants with type 1 diabetes and those with type 2 diabetes are found in Table 2. The clinical characteristics of the non-responders are described in detail in Additional file 1: Table S1.

**Table 1:** Clinical and demographic characteristics of the responders separated by diabetes type and HbA_1c_ level

Variable	Type 1 diabetes						Type 2 diabetes					
	All	HbA1c < 52 mmol/mol (< 6.9%)	HbA1c 52–69 mmol/mol (6.9–8.5%)	HbA1c ≥70 mmol/mol (≥8.6%)	Standardized mean difference, SMD	*p*-value	All	HbA1c < 52 mmol/mol (< 6.9%)	HbA1c 52–69 mmol/mol (6.9–8.5%)	HbA1c ≥70 mmol/mol (≥8.6%)	Standardized mean difference, SMD	*p*-value
Number (%)	1373	284 (20.7)	781 (56.9)	308 (22.4)			1353	725 (53.6)	503 (37.2)	125 (9.2)		
Men, *n* (%)	690 (50.3)	152 (53.5)	391 (50.1)	147 (47.7)	0.077	0.366	822 (60.8)	444 (61.2)	302 (60.0)	76 (60.8)	0.016	0.914
Age, years (SD)	48.6 (16.4)	46.9 (17.0)	49.6 (16.1)	47.8 (16.3)	0.113	0.028	66.6 (9.1)	66.5 (9.1)	66.9 (9.0)	65.5 (9.7)	0.103	0.274
Diabetes duration, years (IQR)	22.0 (12.0–36.0)	19.0 (7.0–32.0)	23.0 (13.0–37.0)	24.0 (13.0–37.0)	0.150	< 0.001	8.0 (4.0–14.0)	6.0 (3.0–11.0)	10.0 (6.0–16.0)	13.0 (6.0–17.0)	0.443	< 0.001
HbA1c mmol/mol (SD)	62 (12.7)						53 (12.5)					
HbA1c, % (SD)	7.8 (1.2)						7.0 (1.1)					
BMI, kg/m^2^ (SD)	26.0 (4.2)	25.2 (3.8)	26.0 (4.2)	26.7 (4.6)	0.239	< 0.001	29.9 (5.3)	29.3 (5.2)	30.3 (5.4)	32.0 (5.5)	0.332	< 0.001
Systolic blood pressure, mmHg (SD)	127.0 (14.0)	124.8 (14.0)	127.5 (13.8)	127.8 (14.2)	0.145	0.009	134.3 (14.3)	134.0 (14.4)	134.5 (13.7)	135.1 (16.5)	0.046	0.687
Antihypertensive medication, *n* (%)	589 (44.7)	99 (36.9)	341 (45.3)	149 (50.2)	0.179	0.006	1070 (80.1)	572 (79.6)	404 (81.9)	94 (76.4)	0.091	0.327
LDL-cholesterol, mmol/L (SD)	2.4 (0.8)	2.5 (0.8)	2.4 (0.8)	2.5 (0.8)	0.077	0.262	2.5 (0.9)	2.5 (0.9)	2.4 (0.9)	2.5 (1.0)	0.026	0.810
Lipid-lowering medication, *n* (%)	642 (48.4)	94 (34.6)	378 (49.8)	170 (57.6)	0.315	< 0.001	900 (68.1)	472 (66.6)	344 (70.1)	84 (69.4)	0.050	0.421
Microalbuminuria, *n* (%)	132 (10.3)	12 (4.6)	70 (9.5)	50 (17.6)	0.285	< 0.001	194 (18.0)	80 (13.9)	83 (20.1)	31 (34.1)	0.323	< 0.001
Macro albuminuria, *n* (%)	31 (2.6)	5 (2.1)	12 (1.8)	14 (5.2)	0.126	0.010	52 (5.0)	27 (4.8)	20 (5.1)	5 (6.1)	0.037	0.884
Estimated Glomerular Filtration Rate, eGFR, mL/min (SD)	90.0 (23.5)	90.6 (20.7)	89.1 (22.6)	91.6 (27.7)	0.071	0.263	82.3 (23.5)	82.5 (22.3)	81.9 (24.0)	83.4 (27.9)	0.038	0.826
Retinopathy, *n* (%)	875 (65.9)	137 (50.6)	520 (68.2)	218 (74.1)	0.333	< 0.001	327 (29.4)	128 (21.7)	153 (36.3)	46 (47.0)	0.366	< 0.001
Coronary heart disease, *n* (%)	83 (6.3)	9 (3.3)	53 (7.0)	21 (7.1)	0.113	0.083	279 (22.4)	136 (20.2)	111 (24.0)	32 (28.6)	0.130	0.081
Stroke, *n* (%)	48 (3.6)	5 (1.9)	32 (4.2)	11 (3.7)	0.093	0.196	96 (7.8)	48 (7.2)	40 (8.9)	8 (7.1)	0.043	0.576
Smoker, *n* (%)	135 (10.1)	14 (5.1)	78 (10.2)	43 (14.4)	0.214	0.001	162 (12.9)	79 (11.7)	58 (12.3)	25 (23.1)	0.203	0.004
Physical activity, daily, *n* (%)	359 (27.6)	90 (33.5)	203 (27.2)	66 (23.2)	0.334	< 0.001	426 (34.9)	251 (38.7)	157 (33.9)	18 (16.7)	0.410	< 0.001
Diabetes treatment					0.136	0.138					0.813	< 0.001
Diet alone, *n* (%)							195 (14.4)	172 (23.7)	19 (3.8)	4 (3.3)		
Oral hypoglycaemic agent alone, *n* (%)							718 (53.1)	419 (57.8)	261 (52.0)	38 (30.9)		
Insulin alone, *n* (%)	1335 (97.2)	271 (95.4)	764 (97.8)	300 (97.4)			130 (9.6)	46 (6.3)	63 (12.5)	21 (17.1)		
Insulin and oral agent, *n* (%)	32 (2.3)	9 (3.2)	15 (1.9)	8 (2.6)			266 (19.7)	76 (10.5)	140 (27.9)	50 (40.7)		
Insulin pump users among insulin users, *n* (%)	356 (26.2)	66 (23.8)	221 (28.5)	69 (22.5)	0.091	0.082	2 (0.5)	1 (0.9)	1 (0.5)	0 (0.0)	0.093	0.729

The descriptive statistics are presented as the means and standard deviations (SD) for normally distributed continuous variables, the median and interquartile range (IQR) for skewed distributions, or number and percentages for categorical variables

**Table 2:** Crude means and standard deviations for the SF-36v2 domains for participants with type 1 diabetes and those with type 2 diabetes

SF-36v2 domains	Type 1 diabetes	Type 2 diabetes
PF	84.4 (21.3)	70.6 (27.0)
RP	80.2 (26.5)	73.6 (29.3)
BP	69.2 (28.0)	62.5 (28.8)
GH	60.3 (23.8)	60.8 (23.7)
VT	54.4 (24.5)	58.8 (24.1)
SF	81.3 (23.9)	79.2 (28.0)
RE	83.2 (24.2)	81.0 (24.8)
MH	72.7 (20.0)	74.6 (21.7)
PCS	50.2 (9.4)	46.5 (9.9)
MCS	48.8 (11.2)	50.8 (11.1)

*PF* Physical functioning, *RP* Role-physical, *BP* Bodily pain, *GH* General health, *VT* Vitality, *SF* Social functioning, *RE* Role-emotional, *MH* Mental health, *PCS* Physical component summary measure, *MCS* Mental component summary measure

In the correlation analysis, the HbA_1c_ level showed weak negative correlations with the SF-36v2 dimensions (− 0.19 to − 0.06) in both type 1 and type 2 diabetes (Table 3).

**Table 3:** Spearman’s rank correlations with *p*-values between SF-36v2 domain scores and glycated haemoglobin (HbA_1c_) level in type 1 and type 2 diabetes

SF-36v2 domain	Type 1 diabetes	Type 2 diabetes
PF	−0.15 (<.0001)	−0.17 (<.0001)
RP	−0.12 (<.0001)	− 0.18 (<.0001)
BP	−0.14 (<.0001)	− 0.13 (<.0001)
GH	−0.19 (<.0001)	− 0.14 (<.0001)
VT	−0.13 (<.0001)	− 0.13 (<.0001)
SF	−0.08 (0.0025)	− 0.12 (<.0001)
RE	−0.08 (0.0056)	− 0.12 (<.0001)
MH	−0.06 (0.0319)	−0.08 (0.0059)

*PF* Physical functioning, *RP* Role-physical, *BP* Bodily pain, *GH* General health, *VT* Vitality, *SF* Social functioning, *RE* Role-emotional, *MH* Mental health

**Fig. 1:**
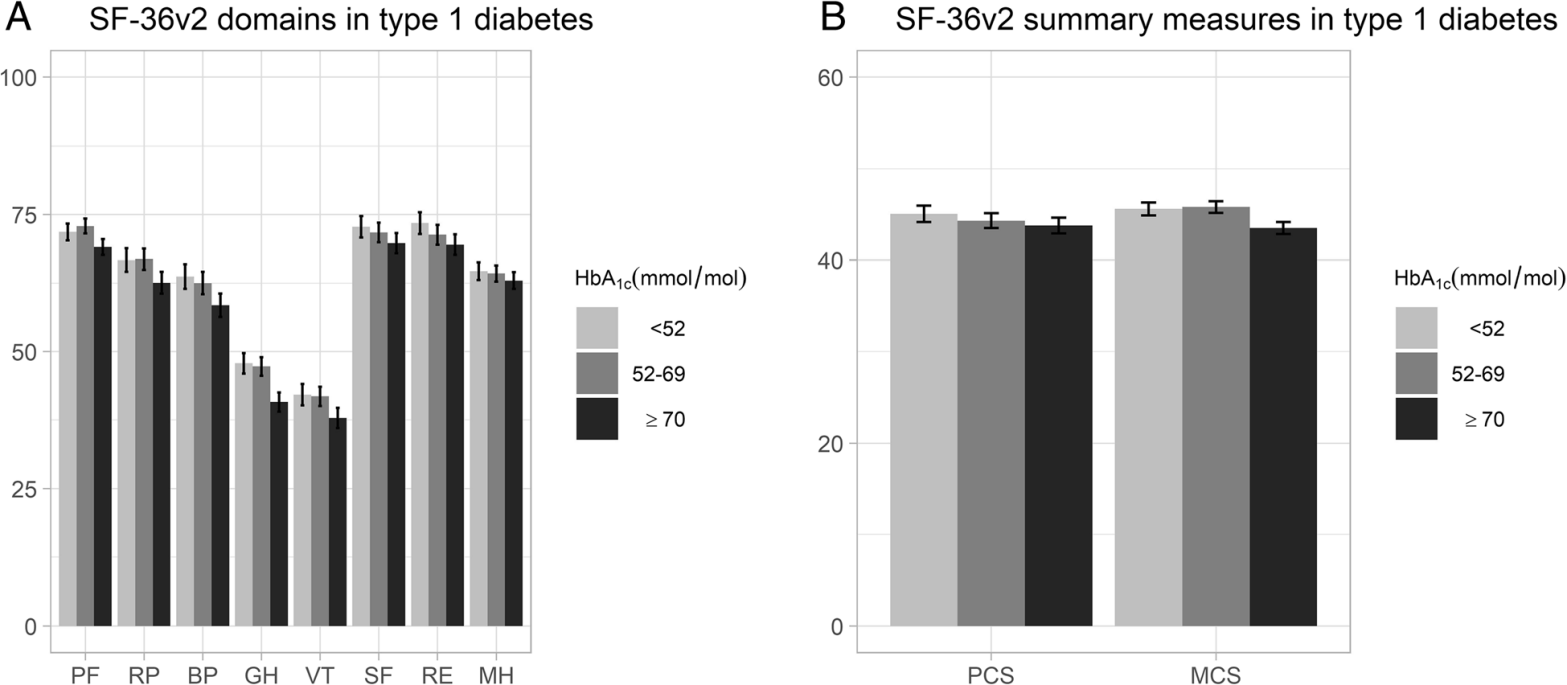
Adjusted regression analyses of HbA_1c_ level and SF-36v2 domains and summary measures in type 1 diabetes. Adjusted least square mean estimates with 95% confidence intervals for SF-36v2 domains (**a**) and for summary measures (**b**) in type 1 diabetes separated by HbA_1c_ level. Adjusted for age, sex, diabetes duration, body mass index, systolic blood pressure, LDL cholesterol level, micro and macro albuminuria, estimated glomerular filtration rate, retinopathy, smoking status, physical activity level, receipt of antihypertensive and lipid lowering treatments, previous coronary heart disease and previous stroke. PF: physical functioning; RP: role-physical; BP: bodily pain; GH: general health; VT: vitality; SF: social functioning; RE: role-emotional; MH: mental health; PCS: physical component summary measure; MCS: mental component summary measure

**Fig. 2:**
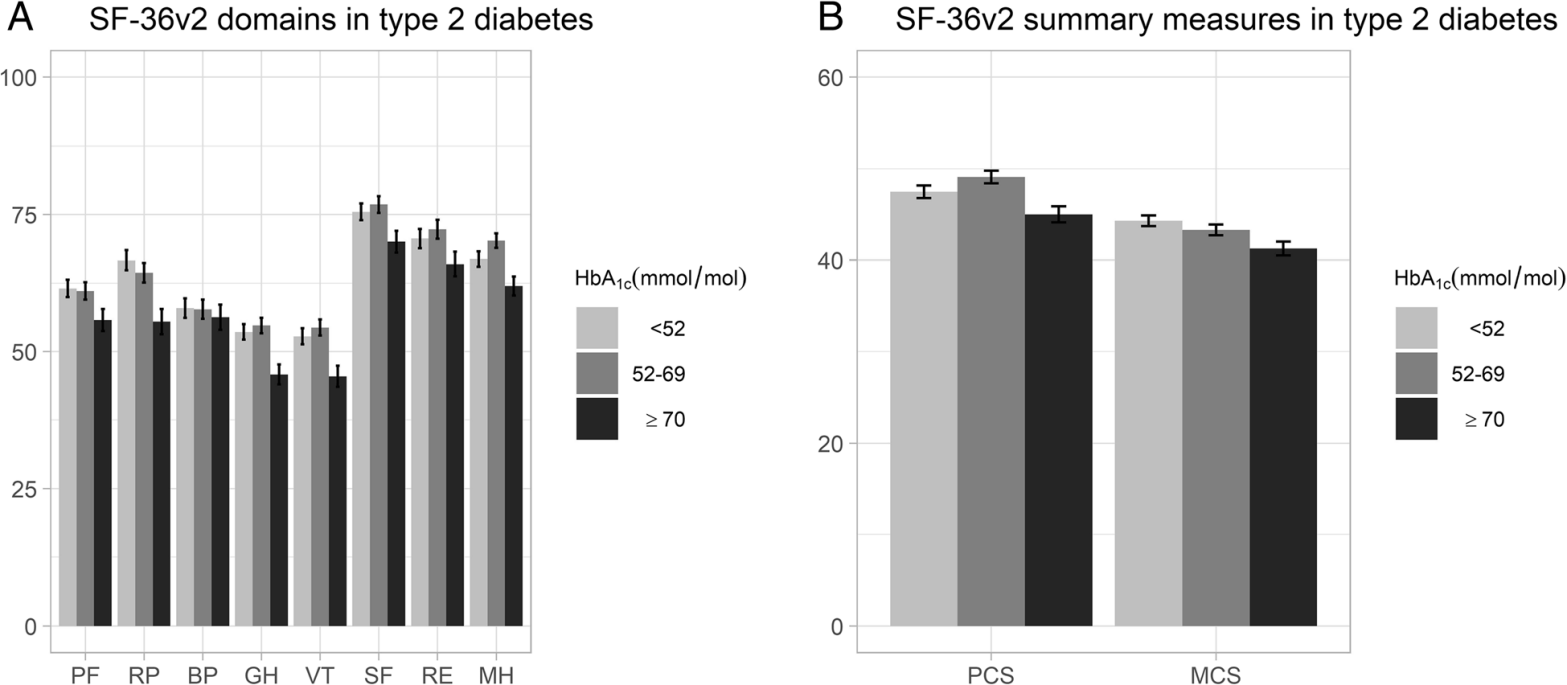
Adjusted regression analyses of HbA_1c_ level and SF-36v2 domains and summary measures in type 2 diabetes. Adjusted least square mean estimates with 95% confidence intervals for SF-36v2 domains (**a**) and for summary measures (**b**) in type 2 diabetes separated by glycated haemoglobin (HbA_1c_) level. Adjusted for age, sex, diabetes duration, body mass index, systolic blood pressure, LDL cholesterol level, micro and macro albuminuria, estimated glomerular filtration rate, retinopathy, smoking status, physical activity level, receipt of antihypertensive and lipid lowering treatments, previous coronary heart disease and previous stroke. PF: physical functioning; RP: role-physical; BP: bodily pain; GH: general health; VT: vitality; SF: social functioning; RE: role-emotional; MH: mental health; PCS: physical component summary measure; MCS: mental component summary measure

The results from the adjusted regression analyses are presented separately for participants with type 1 and type 2 diabetes in Figs. 1 and 2. The detailed least square mean estimates and confidence intervals from the unadjusted and adjusted analyses are provided in Additional file 1: Table S2.

Among those with type 1 diabetes, the adjusted analysis of the HbA_1c_ groups showed that the high-risk group (≥70 mmol/mol/8.6%) had statistically significantly lower means than the well-controlled (< 52 mmol/mol/6.9%) group in five domains (RP, BP, GH, VT, RE), with the largest between-group difference in the GH domain. In the summary components, compared with the well-controlled group, the high-risk group had a statistically significantly lower mean MCS score. There were no statistically significant differences in PCS scores. Both the adjusted PCS and adjusted MCS scores were below the US normal range for all HbA_1c_ groups (Fig. 1, Additional file 1: Table S2).

Among those with type 2 diabetes, the adjusted analysis showed that the high-risk group had the statistically significantly lowest means in all domains except BP. In the MH domain, the sup-optimal group (52–69 mmol/mol/6.9–8.5%) had the statistically significantly highest mean. The clearest differences were observed in the RP, GH, and VT domains. With regard to MCS and PCS, the high-risk group still had the statistically significantly lowest means, which were below the US normal range. The sub-optimal and well-controlled groups were both within the range for PCS but below the range for MCS (Fig. 2, Additional file 1: Table S2).

## Discussion

### Summary of results

With a large randomly selected nationwide sample of adults with type 1 and type 2 diabetes, this study adds current data to the body of research that uses the SF-36 to evaluate HRQOL and assesses its associations with glycaemic control. At the individual level, we observed only weak correlations between scores on the SF-36v2 and glycaemic control. After dividing the samples into three clinically relevant groups according to levels of glycaemic control, the adjusted analyses showed that among participants with type 1 diabetes, compared with the well-controlled group, the high-risk group had statistically significantly lower means in five domains (RP, BP, GH, VT, RE), as well as in the MCS score. For the high-risk group; the largest difference in comparison to the other two groups was seen in the GH domain. In the adjusted analyses for participants with type 2 diabetes, the high-risk group had the statistically significantly lowest means in both summary measures and all domains except BP. The largest differences between the high-risk group and the other two groups were found in the domains RP, GH and VT for participants with type 2 diabetes.

### Comparisons to previous studies in people with type 1 diabetes

For people with type 1 diabetes, we found few previous and no recent studies of HRQOL based on the SF-36 or the RAND-36 and the association of HRQOL with glycaemic control. Compared to the previous studies, our sample size was larger, with a wider age span and a lower mean HbA_1c_ level. Our study adds a new aspect by dividing the sample into three clinically relevant HbA_1c_ groups according to their risk of diabetes complications. The results of a US study including 150 participants support our results, with higher SF-36 scores among those with lower HbA_1c_ levels than among those with higher HbA_1c_ levels [22]. In the present study, GH was the domain with the largest difference among the HbA_1c_ groups, with the lowest score in the high-risk group. This finding is supported by the results in another US study (*n* = 397) that showed that a higher HbA_1c_ level was correlated with worse GH scores [13]. However, in that study, the results were not confirmed in the adjusted analyses [13]. Furthermore, in 2003, no relationship between RAND-36 score and HbA_1c_ level was found in a study conducted in the Netherlands (*n* = 281) [12]. The Diabetes Control and Complications Trial (*n* = 1441) found no differences in SF-36 scores between the intensive insulin treatment group with an HbA_1c_ level of 7.4% (57 mmol/mol) compared to the standard treatment group with an HbA_1c_ level of 9.1% (76 mmol/mol) at the end of the study [11, 35].

### Comparisons to previous studies in people with type 2 diabetes

Among people with type 2 diabetes, previous larger studies using the SF-36 or RAND-36 are more common and include data that are more recent. Some studies divided their sample according to HbA_1c_ level, but the cut-off values for the groups differed from those used in our study. Our results showing lower levels of HRQOL in groups with higher HbA_1c_ levels are supported by SF-36 data from Italy [19] and the ADDITION trial with participants from Denmark, the Netherlands and the UK [17]. The Italian study (*n* = 2499) reported a negative association between the MCS score and the HbA_1c_ level, but after adjustment for hypo- and hyperglycaemic events, this association was no longer significant [19]. In the ADDITION trial (*n* = 1876), participants with HbA_1c_ levels below 7% (53 mmol/mol) had higher PCS and MCS scores than those with higher HbA_1c_ levels [17]. Two studies suggested the lack of an association, namely, a study from the Netherlands (*n* = 1006) that used the RAND-36 and three HbA_1c_ groups [16] and an Estonian study (*n* = 200) that used the SF-36 and divided the sample using a cut-off HbA_1c_ level of 7.5% (58.5 mmol/mol) [15]. The ADDITION and the Italian cohorts are similar to the cohort in our study with regard to age, and the ADDITION cohort had slightly lower HbA_1c_ levels than those in our study [17, 19]. The other studies in which the results were not in agreement with those of our study included younger participants with higher HbA_1c_ levels in comparison to our sample [15, 16]. Among the studies that treated HbA_1c_ as a continuous variable, the results differed. Three studies showed no relationship [17, 18, 20], and two studies supported a relationship [14, 22].

### Complexity

The reasons for the inconsistencies between previous studies and our study could be related to differences in sample characteristics, HbA_1c_ groups, available clinical and demographic data, or reporting results for domains or summary measures. Other reasons could be differences in diabetes care and treatment or their development over time. Norris et al. [9] have also highlighted large differences among studies in a review published in 2011. Norris et al. suggest that the effect of type 2 diabetes on HRQOL might be underestimated because study samples often represent selected groups that are different from the largely heterogeneous type 2 diabetes population. Furthermore, Norris et al. question the use of previously published SF-36 norms for diabetes [9]. As suggested by many, it might also be speculated that the SF-36 is not specific enough with regard to diabetes [4, 9, 10, 23]. Our results add to this complexity based on large heterogeneous samples from a nationwide diabetes register. We found weak correlations between glycaemic control and HRQOL at the individual level. However, when the samples were divided into clinically relevant groups according to levels of glycaemic control, we found that adults with high-risk HbA1c levels have lower levels of HRQOL as measured by the SF-36 in both type 1 and type 2 diabetes.

While there are criticisms of the SF-36, Norris et al. [9] argue that it is important to continue to further research generic HRQOL in people with diabetes using this tool. Given continuous advances in diabetes care, including new medical treatments, technical aids for insulin administration and glucose monitoring, and support for self-management, research results on HRQOL in people with diabetes need to be continuously updated.

It is well known that a high HbA_1c_ level is a risk factor for diabetes complications and death in people with type 1 diabetes and those with type 2 diabetes [36, 37]. In addition, generic HRQOL has been suggested to be a marker for mortality in people with diabetes [26–28]. Our findings show that among people with type 1 diabetes and those with type 2 diabetes, the group with a high-risk HbA_1c_ level has a lower HRQOL. We suggest that the lower HRQOL is not explained by diabetes risk factors or complications, as the results persisted in the adjusted analyses. Our results strengthen the argument that we should focus on these high-risk groups to learn how to further improve their HRQOL and risk factor control. The weak associations on an individual level argues for the need to not focus exclusively on either glycaemic control or HRQOL but rather on both, because both are important parts of the complex life-long challenge of living with diabetes. Another observation is that there are still individuals with well-controlled HbA_1c_ level that have low HRQOL scores, underlining the need to measure HRQOL. A central task for diabetes care and diabetes research is to provide suitable interventions that adequately can support adults with diabetes in their self-management, a desirable glycaemic control as well as a satisfying quality of life. However, the choice of suitable questionnaires to assess quality of life after interventions is a challenge because generic quality of life questionnaires, such as the SF-36, are probably less susceptible for effects on diabetes related problems [28, 38]. Despite that the diabetes care not always can intervene on non-diabetes-specific aspects of HRQOL, it is valuable to increase the awareness of quality of life as interventions related to HRQOL assessments might affect glycaemic control [1, 6, 7].

### Limitations and strengths

Our study has limitations. Owing to the cross-sectional design, causal conclusions cannot be drawn. The analysis was limited to the responders, and the fact that the SF-36 was only offered in Swedish could had resulted in bias, as the proportion of foreign-born individuals might be higher among the non-responders than among the responders. While adding data from a heterogeneous sample, the wide range in age might be a limitation. Despite access to a number of diabetes-related variables, other comorbidities and various other variables not accounted for in our analyses might have influenced the results. The present analyses lack socio-economic data, which is a limitation we will address in future studies. Furthermore, the SF-36 might not capture important diabetes-specific aspects [4, 5, 9, 23]. While generic HRQOL measurements are important, we suggest that it is also important to focus on diabetes-specific aspects to be able to support person-centred care [31, 32, 39]. In our continued work, this is where our main focus will be. Future analyses on the diabetes-specific aspects will provide data on different subgroups, for example regarding age.

The strengths of this study are the updated, large, randomly selected, nationwide sample of people with type 1 diabetes and people with type 2 diabetes and the use of a well-known measure of HRQOL with evidence supporting its validity and reliability. Another strength is the access to clinical and demographic data for both responders and non-responders through the NDR. The responders were representative of the population in the NDR in 2015 for people with type 1 diabetes and those with type 2 diabetes (data on file). Given the 90% coverage rate of the NDR, the results can be deemed representative of the Swedish adult population with diabetes. Our study fills a gap by updating the scores of HRQOL in a broad sample of adults with type 1 and type 2 diabetes.

## Conclusions

This study provides current data in a large nationwide randomly selected sample of adults with type 1 and type 2 diabetes. In people with type 1 diabetes and those with type 2 diabetes, adults with high-risk HbA_1c_ levels have lower levels of HRQOL in most but not all domains of the SF-36v2. The largest differences between the high-risk group and the other two groups was seen in the GH domain for participants with type 1 diabetes and in the domains RP, GH and VT for participants with type 2 diabetes. This was not explained by demographics, other risk factors or diabetes complications. The weak individual-level correlations between HRQOL scores and levels of glycaemic control argues for the need to not focus exclusively on either HbA_1c_ levels or HRQOL scores but rather on both because both are important parts of a complex, life-long, challenging condition.

## Additional file

Additional file 1: **Table S1.** Clinical and demographic characteristics for non-responders separated for type 1 and type 2 diabetes. **Table S2.** Least square mean estimates and 95% confidence intervals for SF-36v2 domains and summary measures in three glycated haemoglobin (HbA_1c_) groups for type 1 and type 2 diabetes. (PDF 190 kb)

## Data Availability

The data that support the findings of this study are not publicly available. The study presented here have been subject to an application to an ethical board and approved for publication related to the specific aim of our research project. With reference to the European General Data Protection Regulation (GDPR), the data are personal data and thereby protected by secrecy.

## Abbreviations

MCS: The Mental Component Summary measure
BP: Bodily pain
GH: General health
HbA_1c_: Glycated haemoglobin
HRQOL: Health-related quality of life
MH: Mental health
NDR: The Swedish National Diabetes Register
PCS: The Physical Component Summary measure
PF: Physical functioning
RE: Role-emotional
RP: Role-physical
SF: Social functioning
SF-36: A self-report questionnaire for generic health-related quality of life, the medical outcomes study health survey short form with 36 items
SF-36v2: The SF-36 version 2
VT: Vitality
