# Supplementary material for: Health-related quality of life and glycaemic control among adults with type 1 and type 2 diabetes – a nationwide cross-sectional study
Source: Health Qual Life Outcomes. 2019 Aug 14;17:141. doi: 10.1186/s12955-019-1212-z (PMC6694672; doi:10.1186/s12955-019-1212-z)
Supplement: Supplementary file 1 — Table S1. Clinical and demographic characteristics for non-responders separated for type 1 and type 2 diabetes. Table S2. Least square mean estimates and 95% confidence intervals for SF-36v2 domains and summary measures in three glycated haemoglobin (HbA1c) groups for type 1 and type 2 diabetes. (PDF 190 kb) [file 12955_2019_1212_MOESM1_ESM.pdf]

Additional file 1

Table S1. Clinical and demographic characteristics for non-responders separated for type 1 and type 2 diabetes

**Variable**	**Non-responders type 1 diabetes** (n=1106)	**Non-responders type 2 diabetes** (n=1116)
Men, %	60.7	58.1
Age, years (SD)	41.2 (15.5)	62.8 (10.9)
Diabetes duration, years (SD)	21.7 (14.0)	8.7 (7.0)
HbA1c, mmol/mol (SD)	65.2 (15.1)	54.7 (14.3)
HbA1c, % (SD)	8.1 (1.4)	7.2 (1.3)
BMI, kg/m^2 (SD)	26.1 (4.6)	30.9 (5.9)
Systolic blood pressure, mmHg (SD)	126.1 (14.0)	134.7 (15.8)
Antihypertensive medication, %	34.6	77.5
LDL-cholesterol, mmol/L (SD)	2.53 (0.79)	2.64 (0.93)
Lipid-lowering medication, %	37.1	60.5
Microalbuminuria, %	11.4	18.4
Macro albuminuria, %	5.6	5.0
Retinopathy, %	66.2	30.5
Smoker, %	15.7	18.4
Physical activity, daily, %	20.3	27.1
**Diabetes treatment**		
Diet alone, %	-	20.1
Oral hypoglycaemic agent alone, %	-	52.5
Insulin alone, %	97.2	8.1
Insulin and oral agent, %	2.3	16.7
Insulin pump users, %	19.9	0.0

Table S2. Least square mean estimates and 95% confidence intervals for SF-36v2 domains and summary measures in three glycated haemoglobin (HbA_{1c}) groups for type 1 and type 2 diabetes

**SF-36v2 domain and summary measure**	**Type 1 diabetes**						**Type 2 diabetes**					
	Unadjusted analysis			Adjusted analysis			Unadjusted analysis			Adjusted analysis		
	HbA1c <52 mmol/mol (<6.9%)	HbA1c 52-69 mmol/mol (6.9-8.5%)	HbA1c ≥70 mmol/mol (≥8.6%)	HbA1c <52 mmol/mol (<6.9%)	HbA1c 52-69 mmol/mol (6.9-8.5%)	HbA1c ≥70 mmol/mol (≥8.6%)	HbA1c <52 mmol/mol (<6.9%)	HbA1c 52-69 mmol/mol (6.9-8.5%)	HbA1c ≥70 mmol/mol (≥8.6%)	HbA1c <52 mmol/mol (<6.9%)	HbA1c 52-69 mmol/mol (6.9-8.5%)	HbA1c ≥70 mmol/mol (≥8.6%)
PF	88.15 (87.37-88.93)	84.68 (84.21-85.15)	80.21 (79.45-80.96)	71.79 (70.27-73.31)	72.88 (71.50-74.26)	69.04 (67.61-70.48)	73.89 (73.27-74.51)	68.58 (67.83-69.33)	60.17 (58.69-61.66)	61.53 (59.94-63.11)	61.08 (59.50-62.66)	55.77 (53.75-57.79)
RP	83.97 (83.00-84.94)	80.77 (80.18-81.36)	75.31 (74.37-76.25)	66.69 (64.57-68.82)	66.84 (64.92-68.76)	62.56 (60.57-64.55)	77.91 (77.24-78.58)	70.88 (70.07-71.69)	59.26 (57.63-60.89)	66.66 (64.86-68.46)	64.39 (62.60-66.17)	55.46 (53.16-57.76)
BP	74.57 (73.54-75.59)	69.37 (68.75-69.99)	63.62 (62.63-64.61)	63.68 (61.43-65.94)	62.50 (60.46-64.54)	58.47 (56.36-60.59)	65.36 (64.70-66.03)	60.14 (59.35-60.94)	55.24 (53.63-56.84)	57.96 (56.18-59.74)	57.73 (55.97-59.49)	56.28 (54.00-58.57)
GH	65.45 (64.59-66.31)	61.36 (60.84-61.88)	52.73 (51.91-53.56)	47.87 (45.99-49.74)	47.28 (45.58-48.98)	40.78 (39.01-42.54)	63.24 (62.70-63.78)	60.11 (59.46-60.75)	49.39 (48.09-50.68)	53.60 (52.17-55.02)	54.77 (53.36-56.18)	45.82 (43.99-47.66)
VT	57.91 (57.01-58.80)	55.36 (54.82-55.90)	48.75 (47.88-49.61)	42.12 (40.17-44.06)	41.80 (40.03-43.57)	37.84 (36.00-39.67)	60.84 (60.29-61.40)	58.37 (57.71-59.04)	48.63 (47.30-49.97)	52.79 (51.31-54.28)	54.39 (52.92-55.86)	45.48 (43.57-47.39)
SF	84.49 (83.61-85.36)	81.44 (80.91-81.96)	77.81 (76.96-78.65)	72.74 (70.77-74.70)	71.69 (69.91-73.46)	69.76 (67.91-71.60)	82.94 (82.37-83.51)	80.28 (79.59-80.96)	72.00 (70.62-73.39)	75.46 (73.92-77.00)	76.80 (75.27-78.32)	70.02 (68.04-72.00)
RE	86.05 (85.16-86.94)	83.37 (82.83-83.90)	80.27 (79.41-81.13)	73.42 (71.43-75.40)	71.28 (69.48-73.08)	69.48 (67.61-71.34)	81.43 (80.78-82.08)	78.69 (77.91-79.47)	68.01 (66.43-69.59)	70.59 (68.84-72.34)	72.28 (70.54-74.02)	65.96 (63.73-68.20)
MH	74.42 (73.68-75.15)	73.00 (72.55-73.44)	70.43 (69.71-71.14)	64.66 (63.05-66.27)	64.25 (62.79-65.71)	62.97 (61.46-64.49)	75.36 (74.86-75.86)	75.72 (75.12-76.32)	65.10 (63.89-66.32)	66.87 (65.51-68.22)	70.21 (68.87-71.56)	61.97 (60.22-63.72)
PCS	49.77 (49.36-50.18)	48.98 (48.73-49.23)	47.58 (47.18-47.97)	45.07 (44.16-45.97)	44.31 (43.49-45.14)	43.78 (42.92-44.63)	51.21 (50.96-51.47)	51.33 (51.02-51.64)	46.00 (45.37-46.63)	47.47 (46.77-48.17)	49.09 (48.40-49.78)	45.01 (44.11-45.90)
MCS	52.24 (51.89-52.58)	50.45 (50.24-50.66)	47.74 (47.41-48.08)	45.60 (44.89-46.31)	45.81 (45.16-46.45)	43.51 (42.84-44.18)	48.04 (47.81-48.27)	45.23 (44.95-45.50)	42.69 (42.13-43.25)	44.30 (43.71-44.90)	43.30 (42.71-43.90)	41.24 (40.49-42.00)

PF: physical functioning; RP: role-physical; BP: bodily pain; GH: general health; VT: vitality; SF: social functioning; RE: role-emotional; MH: mental health; PCS: physical component summary measure; MCS: mental component summary measure.
